# Deterioration of lung function and its association with eosinophil count

**DOI:** 10.6026/97320630019644

**Published:** 2023-05-31

**Authors:** Bama Rajanayagam, Sundaramahalingam Manikandan, Nag Anand

**Affiliations:** 1 Bharath Institute of Higher Education and Research, Selaiyur, Chennai, India; 2 Department of Physiology, Indira Medical College and Hospital, Thiruvallur, Tamilnadu, India; 3 Department of Physiology, Tagore Medical College and Hospital, Rathinamangalam, Chennai, India; 4 Department of General Medicine, Sree Balaji Medical College and Hospital, Chromepet, Chennai, India

**Keywords:** lung function, eosinophil count, infections

## Abstract

Exposure to lethal dust particles has a negative impact on human's health. This work investigated the association between respiratory symptoms and eosinophil levels between quarry workers and the controls. A total of 75 workers exposed to quarry dust and 45 age, sex, body mass index-matched unexposed controls participated in this study. Results of this study indicated that the quarry dust particles produced inflammatory responses, increasing the mean eosinophil level (7.56 ± 2.94), causing allergic respiratory symptoms such as rhinitis , chest tightness, wheeze, sputum production thus impairing the lung function with decline in FEV1(80.84 ±108.8) level in workers exposed to quarry dust compared to controls (P≤0.00**) Conclusion: The presence of increased levels of respiratory irritants in quarry sites may explain the higher prevalence of respiratory infections and exacerbated inflammatory reactions found to ascertain the link between increased eosinophil count and the lung impairment in quarry workers.

## Background:

Large-scale urbanization and industrialization in the nation have contributed to environmental degradation, pollution, and occupational threats. Health risks are caused by pollution, and it is estimated that 17% of occupational diseases worldwide and 18% of occupational disease-related deaths occur in India. There is a common misperception that industries and industrialized nations are the main focus of occupational health. However, millions of people work in industries like stone grinding, quarry work, weaving, etc. in a country like India. In regions rich in natural resources like marble, limestone, and gypsum, the art of quarrying has been practiced for centuries. As a result of inhaling airborne particulates, quarry workers are exposed to a variety of risks that are very dangerous to their health and safety [8]. Working in the stone quarrying and related mining industries has been linked to numerous negative health and social effects. Due to the numerous processes and operations, such as stone cutting, loading, and crushing, the stone quarrying industry is known to have relatively high rates of respiratory morbidity [2]. The detrimental effects of quarrying activities include soil erosion, the creation of swamps, the degradation of groundwater, noise from rock blasting, the emission of dust, smoke, and fumes, the production of toxic gasses, and ground vibration [3]. A specific risk in some quarries is inhaling silica-containing dust, which can cause silicosis. According to studies, quarry dust exposure increases the incidence of lung cancer, pulmonary TB, and airway disorders among workers. Workers who are exposed to dust are most at risk for respiratory issues and occupational lung diseases, which are also the leading causes of morbidity and mortality worldwide [4].

A significant and important aspect in evaluating the state of the respiratory system is pulmonary function testing. Numerous studies have been conducted so far to determine the effect of pollution on lungs. According to the findings, increased particulate matter exposures negatively impact lung function. Numerous earlier studies have documented how lung function gradually declines with increased silica dust exposure time [9-10, 11]. According to certain research, workers exposed to silica dusts at even low levels over extended periods of time may experience lung function issues [6]. FEV1, or forced expiratory volume, is a crucial indicator of lung volume. It is a predictor among the particular occupational group of desert-based sandstone quarry employees. Allergic reaction is unique due to increased activation of particular white blood cells termed mast cells and basophils by an antibody [1]. Eosinophilia in the peripheral blood is typically considered a sign of allergies. Numerous studies have shown a connection between peripheral blood eosinophil levels and pulmonary function impairment [5]. Quarry employees experience a variety of allergy reactions brought on by dust, which can result in eosinophilia. The pulmonary function tests (PFT) and the differential count of leucocytes were carried out among the workers by standard technique. A decrease in pulmonary function test values was noted with the increment of blood eosinophil level. The goal of the current study was to assess the pulmonary function deficits brought on by an increase in peripheral blood eosinophil in quarry workers.

## Material and Methods:

## Study design procedure:

A detailed clinical examination was done by the supervision of physician to conduct a comprehensive study. The weight of the participants was recorded in kilograms using a standard weight scale and height was recorded using height scale. The number of participants examined per day was 6. The first step involved was a basic clinical screening by the physician, which would be followed by a brief explanation of the procedures involved in the study to the subjects.

## Ethical clearance and consent form:

Ethical clearance was duly obtained from the Institutional Human Ethical Committee NO: 19 January/ 2021, and verbal informed consents were taken from both groups.

## Specifications of participants for exposed group:

The study was conducted at stone quarries from Yelaanjeri village, Vempakkam Vattam, which is located at Thiruvannamalai district, 120 Km away from Chennai. It is a small village with numerous stone quarries flanged on all sides of the roads, and almost the entire population depends on quarry industries for their livelihood. The workers selected for this study were aged between 20-55 years, were all male participants, and had not less than 2 years of work exposure.

## Specifications of participants for control group:

A 45 healthy volunteers, not exposed to quarry dust, matched in terms of age, sex, gender and socio-economic status with that of exposed participants, were included as control group participants. They are residing in an area free of air pollution due to quarrying or any other form of industries or factories. The control group subjects from an area called Allikuzhi village, Thiruvallur District, Tamilnadu where no quarries are present in and around 20 km and have no exposure to quarries.

## Inclusion and exclusion Criteria for participants:

Age 20 to 55 years, not exposed to quarry or any other form of industrial air pollution, and willing participants in this study were recruited as control participants.

## Exclusion Criteria for Control Group Participants:

The control subjects who had no history of respiratory dysfunction, or signs of liver, heart, generic, haematological, and bone diseases were excluded from the study.

## Sample size:

The study was carried out on 120 study participants, obtained by using the formula (see PDF)

Substituting in the above formula, the minimum required sample size (n) is 118, which is rounded to 120 [12].

## Pulmonary Function Test (Spirometer):

A BOLD structured questionnaire was used to assess respiratory problems, and qualified physicians performed medical examinations (health history and physical examination). A suspended particulate meter was used to quantify the concentration of particles in the air (mg/m3). Readings were obtained at four separate locations (cardinal directions) of the quarry site, key activity regions in order to arrive at a mean value for a single sample location. The very next day, after the basic clinical examination and recruitment of the study participant, spirometry was conducted under the supervision of the physician to find out the FEV1, values by using a portable spirometer (RMS Helios 401). Spirometry was done between 10 am to 1 pm in order to avoid diurnal variation. The subjects were motivated to give their best effort during the procedure. After each trial, the subject was given 2 minutes of rest. Three readings were taken. Each test was signed, dated and a comment was made on the patient's effort by the physician.

## Sample Collection and Analysis of haematological indices:

The study was conducted during the months of February 2022 to July 2022. Prior consent and self-designed questionnaires was administered to all the subjects by reading and explaining the purpose of the work. The blood samples were collected from the quarry site and control site during midmorning to noon (10.00 am to 12.00 pm) for both the subjects by venepuncture and collected with both EDTA sample bottles and non-EDTA or plain bottles. The blood samples were stored under cold temperature and later analysed for eosinophil counts using Auto-Analysers (Beckman Coulter, model number ACTDIFF-02) at Hi-tech Diagnostic centre, Kanchipuram.

## Statistical Analysis:

Descriptive data are expressed as Mean± SD. The statistical analysis carried out for comparative analysis includes independent t-Test and Univariate analysis by SPSS 21.0. The statistical significance was calculated p < 0.05* and p<0.001 respectively.

## Result:

There were no significant differences in age, height, body weight, or BMI between the test individuals exposed to quarry pollutants and their unexposed controls, according to demographic and baseline data (Table 1).

The mean respirable dust concentration was greater than the Occupational Safety and Health Administration's (OSHA) allowed standard of 5 mg/m3 at each of the sample sites that were chosen. The present data showed, the average respirable dust concentrations at the different work site drilling (6.16± 0.52) crushing (6.48± 0.16), loading (5.21± 30), administration (5.16± 0.34) were significantly higher (P≤0.001**) than the average concentrations at the control site (0.91 ±0.11) (Table 2). Additionally, the hazard quotients of the respirable dust in all of the chosen locations were >1, suggesting a potential negative impact on pulmonary health.

In (Table 3), subjects exposed to quarry pollutants had symptoms like chest discomfort (22%), breathlessness (17.3%), runny nose (42%), persistent cough (24%), sneezing (33.3%), and wheezing that were significantly more common (P<0.001) than they were in the unexposed control group. In test participants and controls, frequent sneezing, runny nose, coughing, and wheezing were the most common respiratory symptoms.

## Discussion:

Quarrying is an excavation process that involves extraction methods for utility purposes which causes environmental pollution. Inhaling these quarry dust while working on a processing line has an adverse effect on workers' health as well as the environment and society. According to reports, breathing in dust particles can cause lung damage and respiratory issues in humans by lodging in their lungs [13,14].The impact of dust particle on the respiratory system depends on where the dust settles and how the respiratory system reacts to the inhaled particle .For example, irritating dust in the nose can cause rhinitis, an inflammation of the mucous membrane, Inflammation of the trachea (tracheitis), bronchi (bronchitis), or alveoli (alveolitis) may be observed if the particle affects the bigger air passageways [16]. This study was carried out to find the association between lung function and eosinophil count, which showed increased eosinophil count and lower FEV1 value, indicating the deterioration of lung function. It was found that the incidence of respiratory symptoms of frequent sneezing, wheezing and unproductive cough were significantly higher among the quarry workers exposed to pollutants, compared to their control population. A study has also reported higher prevalence rate of specific respiratory symptoms of dry cough (7.28%), sneezing (11.52%), chest pain (8.09%) and breathlessness (2.7%) among workers exposed to dust emitted from crushing of granite when compared to the controls [15]. It has been reported that, 3.2 million people has died from chronic obstructive pulmonary disease (COPD) in 2015, although there are already more than 300 million asthmatics in the world. Eosinophil levels in the peripheral blood have been found to be correlated with pulmonary function impairment in studies [17-18, 19]. Eosinophilic airway problems encompass a group of pathologies characterized by increased levels of eosinophil in the blood and tissues. Increased levels of eosinophil have been linked to allergic reactions. Similarly, patients positive for nerve growth factor have been reported to have a significantly higher number of eosinophil, which play an important role in allergies and respiratory diseases [20]. Eosinophilic airway inflammation and airway remodelling leading to persistent airflow obstruction are characteristic features of asthma, but the link between them is unclear [21]. Eosinophil functions in a wide range, homeostatic and pathological, mediated by cell surface receptors and specific secretory granules that interact with their microenvironment. Development, differentiation, activation and survival of eosinophil are regulated by type 2 cytokines, including interleukin (IL)-5, the main cause for eosinophilopoiesis. Interleukin (IL)-4, IL-5 and IL-13, plays a vital role in the pathophysiology of eosinophilic airway diseases such as asthma, chronic rhinosinusitis, eosinophilic granulomatosis with polyangiitis and hypereosinophilic syndrome [22]. Studies have reported the association of blood eosinophils with lower FEV1 in asthmatic patient and a significant decline in FEV1 in COPD patients [5]. However, there is apaucity on how the eosinophils increases with an increased decline in the lung function. Clinical, diagnosis is important for airway illnesses for direct therapeutic decision. Evaluation of airway problems is done using spirometry, which is used to measure airflow during forced expiration. This test is crucial for the identification of COPD and has a significant effect on the treatment of asthma. Blood eosinophil levels are another biomarker for airway disease and for eosinophilic airway Inflammation. This study shows an elevated count of eosinophils with an increase in the decline of the values FEV1.The allergic manifestations that has been noticed in our study are symptoms such as sputum production, running nose, persistent cough, wheezing, chest tightness that were comparatively significant in quarry workers could be due higher level of inhaled respirable dust in the quarry site. Olusegun et al. and Nwibo et al., [3,4] reported a high prevalence of catarrh or "runny nose" and cough (26%, 40.5% and (20%, 26%) in the respondents exposed to granite dust in Calabar and Abeokuta, Nigeria respectively. Earlier research are consistent with their findings that showed a high eosinophil level as well as greater prevalence of respiratory symptoms such as sputum production and cough among the test subjects, which may be due to aggravated inflammatory responses to exposure to quarry pollutants [23]. Similarly, an elevated blood eosinophil count of ≥300 cells/L or more is linked to undiagnosed structural airway abnormalities and is a risk factor for rapid lung function decline in older persons. Backman [20] has reported higher level of eosinophils to be related to an annual decline of 27ml in FEV1 in a mean follow up time of 18 years of study. The present study showed significant increase in eosinophil count correlated with decrease in FEV1 suggested that quarry dust exposure increases blood eosinophil level, which is associated with obstructive pulmonary function impairments.

## Conclusion:

Exposure to air pollutants from quarry site increases the eosinophil counts due to aggravated inflammatory responses with corresponding decrease in the values of FEV1. This result suggests that prolonged exposure could deteriorate the pulmonary function of the quarry workers resulting in obstructive pulmonary diseases. This could be prevented by educating the quarry workers the importance of the usage of PPE, by regular screening and health check-ups, by monitoring and regulating the levels of air pollutants at the quarry site and by implementing awareness programs regarding exposure and the consequences of quarry dust.

## Figures and Tables

**Figure 1:**
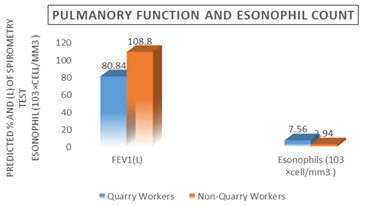
shows that the pulmonary function test of FEV1 (80.84± 108.8), and the mean eosinophil count (7.56±2.94 103 cells/mm3) of the quarry workers was substantially higher (P ≤0.01) than that of the controls.

**Table 1:** Demographic features for the study group and control group

	**Study Group (n=75)**		**Control Group (n=45)**	
	Mean	±Std. Deviation	Mean	±Std. Deviation
Age (Years)	35.43	±8.19	38.64	±7.59
Height (Cms)	164.05	±5.76	169.93	±6.79
Weight (Kgs)	64.96	±13.24	74.71	±9.46
BMI	24.1	±4.60	25.99	±3.79

**Table 2:** Mean levels of respirable dust (mg/m3) at selected locations of the quarry site compared with the control site

**Work site**	**Respirable dust (mg/m3)**	**Hazard quotient**
Drilling	6.16±0.52*	1.14
Crushing	6.48±0.16**	1.32
Loading	5.21±0.30*	1.08
Administrative office	5.16±0.34*	1.01
Control site	0.91±0.11	0.12
*Significantly higher than control (P<0.001). OSHA permissible level=5 mg/m3, OSHA: Occupational Safety and Health Administration

**Table 3:** Comparison of the frequency of infective indices of exposure to quarry pollutants between the control and quarry workers

**ClinicalSymptoms**	**Control**	**%**	**Quarryworkers**	**%**	**χ²**	**Pvalue**
	**N(45)**		**N75**			
Sputum	12	26.66667	37	49.33333	48.17	0.000**
Runningnose(Catarrh)	18	40	24	32	68.03	0.011*
Constantsneezing	6	13.33333	25	33.33333	57.45	0.000**
Continuouscough	8	17.77778	18	24	39.21	0.000**
Wheezing	6	13.33333	20	26.66667	27.01	0.001**
Chestdiscomfort	5	11.11111	17	22.66667	18.43	0.001**
Breathlessness	7	15.55556	13	17.33333	31.91	0.021*
